# Invasion and MMP expression profile in desmoid tumours

**DOI:** 10.1038/sj.bjc.6601661

**Published:** 2004-03-23

**Authors:** H Denys, O De Wever, B Nusgens, Y Kong, R Sciot, A-T Le, K Van Dam, A Jadidizadeh, S Tejpar, M Mareel, B Alman, J-J Cassiman

**Affiliations:** 1 Center for Human Genetics, University of Leuven, Leuven, Herestraat 49, B-3000 Leuven, Belgium; 2 Laboratory of Experimental Cancerology, Department of Radiotherapy and Nuclear Medicine, Ghent University Hospital, De Pintelaan 185, B-9000, Belgium; 3 Laboratory of Connective Tissues Biology, Tour de Pathologie, University of Liège, Sart Tilman, Belgium; 4 The Program in Developmental Biology, The Hospital for Sick Children and the University of Toronto, 555 University Avenue, Toronto, Ontario, Canada M5G1X8; 5 Laboratory of Morphology and Molecular Pathology, University of Leuven, Minderbroedersstraat 12, B-3000 Leuven, Belgium

**Keywords:** MMP expression, desmoids, invasion

## Abstract

Desmoid tumours are locally invasive soft tissue tumours in which *β*-catenin mediated TCF-dependent transcription is activated. The role of soluble factors secreted by the myofibroblastic desmoid tumour, which could stimulate tumour invasiveness, was investigated. Using collagen gel invasion assays, the presence of factors stimulating invasion in desmoid conditioned media (CM) could be established. Since matrix metalloproteinases (MMPs) have been implicated in the process of tumoral invasion, the expression levels of the MMP family members were evaluated. Quantitative reverse transcription–PCR was used to determine the expression levels of MMP1, MMP2, MMP3, MMP7, MMP11, MMP12, MMP13, MMP14 and the inhibitors TIMP1, TIMP2 and TIMP3. Besides overexpression of MMP7, a known TCF-dependent target gene, a striking upregulation of the expression levels of MMP1, MMP3, MMP11, MMP12 and MMP13 in desmoid tumours, compared to unaffected fibroblasts from the same patients, was found. Treating the CM of desmoids with a synthetic and a physiologic MMP inhibitor reduced the invasion-stimulating capacity of the desmoid CM by approximately 50%. These results suggest the involvement of soluble factors, released by the desmoid cells, in stimulating invasion and implicate the MMPs as facilitators of invasion.

Desmoid tumours (also called aggressive fibromatosis) are locally invasive, benign soft tissue tumours, composed of a clonal proliferation of fibroblast-like cells with characteristics of myofibroblasts. They can occur as sporadic lesions or as part of familial adenomatous polyposis, which is caused by germline mutations in the adenomatous polyposis coli (APC) gene. Sporadic desmoids harbour somatic mutations in either the APC gene or in the *β*-catenin gene, resulting in *β*-catenin protein stabilisation (Li *et al*, 1988; Tejpar *et al*, 1999). Previously, we demonstrated constitutive TCF activation in primary desmoid cell cultures and showed that *β*-catenin binds and activates TCF-3 in these tumours (Tejpar *et al*, 2001).

Although desmoids are classified as benign tumours, they can be very invasive and aggressive. They invade surrounding viscera and neurovascular structures, which may cause significant morbidity and mortality (Lewis *et al*, 1999). The aggressive growth pattern and their tendency for recurrence make these tumours difficult to treat. As desmoids are locally invasive lesions, cells from the tumour may need to produce factors in order to promote invasion into the extracellular matrix (ECM).

Tumour invasion is a complex process that requires interaction between the invasive cells and the ECM. A crucial step in invasive processes is the proteolytic degradation of the ECM and basal membranes (Curran and Murray, 1999). Among the enzymes responsible for ECM degradation, several studies have shown a critical role played by matrix metalloproteinases (MMPs) (De Clerck *et al*, 1992). Matrix metalloproteinases are zinc-dependent endopeptidases, which are collectively capable of degrading all constituents of the ECM. Based on their structure and substrate specificity, they can be divided into subgroups of collagenases, stromelysins, gelatinases, membrane-type MMPs and other MMPs.

In this study, we will show the presence of invasion-stimulating factors in desmoid conditioned medium (CM) and we will examine the MMP expression in desmoids and fascia samples. Besides overexpression of MMP7, a known *β*-catenin-dependent target gene in colon cancer, the results show an overexpression of MMP1, MMP3, MMP11, MMP12 and MMP13 in desmoid tumours compared to normal fibroblasts (fascia tissue). Using MMP inhibitors in invasion assays, we will show that MMPs produced by the desmoid tumours facilitate invasion.

## MATERIALS AND METHODS

### Reagents

The noncytotoxic broad-spectrum MMP inhibitor, Galardin, also called GM6001 or Ilomastat, was dissolved in DMSO and was kindly provided by Dr Kjeld Danø (The Finsen Laboratory, Copenhagen, Denmark or purchased from Raylo Chemicals, Edmonton, Canada). An inactive structural analogue of Galardin was dissolved in matched volumes of DMSO and served as negative control (Calbiochem, La Jolla, CA, USA). The serine proteinase inhibitor Aprotinin was purchased from ICN Pharmaceuticals (Costa Mesa, CA, USA), recombinant TIMP1 was from R&D Systems (Abingdon, UK). Mouse anti-human MMP7 monoclonal antibody was bought from Chemicon (Hofheim, Germany). Soluble type I collagen, derived from rat-tail, was purchased from Upstate Biotechnology (Lake Placid, NY, USA).

### Cell cultures

Primary cell cultures of seven desmoid tumours and their normal fascia controls (patients consented for the use of their tissue) were derived by collagenase treatment of tissue biopsies and grown in Dulbecco's modified Eagle's medium (DMEM) (Life Technologies, Ghent, Belgium) supplemented with 10% fetal calf serum (FCS). All studies were performed using cultures in passage two.

To prepare serum-free CM, cells were harvested by trypsinisation, counted and 1 × 10^6^ cells were cultured in DMEM with 10% FCS in T-75 culture flasks (BD Falcon, Sint-Niklaas, Belgium). After 24 h, cells were washed three times with phosphate-buffered saline, incubated with serum-free medium for 12 h, followed by change of medium and incubation in 6 ml serum-free medium. This 6 ml of CM was collected 48 h later, centrifuged and stored at −70°C until use, a procedure that did not alter its biological activity as tested with 6 months old CM. Conditoned medium of fibroblasts or desmoid was 5 × concentrated in centriprep tubes YM-10 (Amicon, Millipore Corp., Bedford, MA, USA) and passed through a filter with a 0.22 *μ*m pore size, before testing its invasion-stimulating capacity.

HCT-8/E11 (E, epithelioid morphotype) is a cloned human colon cancer cell line cultured as described earlier (Vermeulen *et al*, 1995).

The LoVo cell line was purchased from the American Type Culture Collection (ATCC, Rockville, MD, USA; CCL 229). The CCL 229 cell line is derived from a metastatic nodule in the left supraclavicular region of a 56-year-old patient with histologically proven diagnosis of adenocarcinoma of the colon (Drewinko *et al*, 1976).

### Sulphorhodamine B assay

Sulphorhodamine B assay according to Skehan's method was used to examine cell toxicity (Skehan *et al*, 1990). Experiments were run in octoplet, and were repeated twice.

### Invasion assays

Invasion assays were performed according to Bracke *et al* (2001). Briefly, six-well plates were filled with 1.25 ml of neutralised type I collagen solution and polymerised at 37°C. A measure of 500 *μ*l of 5 × concentrated CM of fascia or desmoid cultures of equal cell numbers together with 500 *μ*l of medium containing 1 × 10^5^ HCT-8/E11 cells were seeded on top of the collagen and incubated at 37°C during 24 h. Invasion was measured using an inverted microscope controlled by a computer program. Invasive and superficial cells were counted in 10 fields of 0.157 mm^2^. The invasion index was expressed as the percentage of cells invading the gel over the total number of cells added. None of the agents tested in the 24 h invasion assay interfered with viability (Trypan blue exclusion test). Invasion chambers were prepared by coating 10 mm tissue culture inserts with 8 *μ*m polycarbonate membrane-modified Boyden chamber inserts (Becton-Dickinson) with 85 *μ*g/cm^2^ Matrigel (Becton-Dickinson). In a similar manner, 1 × 10^5^cells derived from desmoid tumours were cultured onto the top chamber of invasion chamber with and without the addition of Ilomastat. The cells were allowed to migrate across the membrane for 24 h. The Matrigel and cells were removed from the top half of the membrane, and number of cells crossing the membrane was averaged over 10 high-powered fields (hpf's) counted, after staining the cell nuclei with 4,6-diamidino-2-phenylindole (DAPI).

### Quantitative reverse transcription (RT)–PCR

Total RNA was extracted from primary desmoid and fascia cultures using the RNeasy kit (Qiagen). Reverse transcription –PCR for all MMPs (except for MMP7) and TIMPs was carried out as follows. The mRNA of interest and the 28S rRNA were quantified by RT–PCR using 10 ng of total RNA. For each mRNA, the sequences of primers, the optimal conditions of reaction (number of amplification cycles and temperatures) and the amount of primers and internal standards were used as previously described (Nusgens *et al*, 2001; Lambert *et al*, 2001), in order to be within the linear range of measurement under noncompetitive conditions. The efficiency of the RT and the amplification reactions was monitored by adding in each tube an appropriate number of copies of a synthetic RNA (sRNA) that can be reverse-transcribed and amplified with the primer pair used for the endogenous mRNA of interest, but giving rise to a product slightly larger or smaller than the product amplified from mRNA, allowing its discrimination after migration in a 10% polyacrylamide gel. After migration, the gels were stained with Sybergreen (Biorad, Hercule, CA, USA) or Gelstar (FMC Bioproducts, Rockland, ME, USA) and the intensity of the fluorescent signals was measured in a fluor-S-MultiImager (Biorad). Each measurement was normalised to the cotranscribed and coamplified internal standard. The normalised values were expressed in arbitrary units (AU) per unit of 28S rRNA measured in the same dilution of RNA to correct for RNA input of each sample. For negative controls, RT–PCR was performed without cellular RNA.

Quantitative RT–PCR for MMP7 was carried out by ABI Prism 7700 (Applied Biosystems). Probes and primers for MMP7 and porphobilinogen deaminase (PBGD), the internal control, were designed by Primer Express 1.0 (Applied Biosystems). The probes were labelled on the 5′ end by FAM and 3′ end by TAMRA. The primer sequences for MMP7 and PBGD are available on request. TaqMan master mix kit from Eurogentec was used and the general PCR conditions were applied as recommended by Applied Biosystems. Standard curves for MMP7 and PBGD were drawn by Excel (Microsoft) upon the threshold cycle (Ct) values and the relative concentrations of the standards and the relative concentrations for desmoid and fascia samples were calculated from the detected Ct values and the equation of the curves. The values obtained for MMP7 were divided by the values of PBGD to normalise for differences in RT.

### Casein zymography

Conditioned medium obtained from equal cell numbers was concentrated 25 × and 20 *μ*l was treated with nonreducing sample buffer without boiling. Tris-Glycine polyacrylamide precast gel electrophoresis was performed using a 12% polyacrylamide gel containing 0.05% casein (Invitrogen, Merelbeke, Belgium). After electrophoresis, gels were washed for 2 × 30 min in 2% Triton X-100 solution at room temperature and incubated overnight at 37°C in MMP substrate buffer (50 mM Tris-HCl, pH 7.5, 10 mM CaCl_2_). Gels were rinsed again in distilled water, stained with 0.5% Coomassie brilliant blue R-250 in 40% methanol and 10% acetic acid for 20 min, and destained with 40% methanol and 10% acetic acid. Proteolytic activities appeared as clear bands of lysis against a dark background of stained casein. To verify that the detected caseinolytic activities were specifically derived from MMPs, the gels were incubated in the MMP substrate buffer with 10 mM EDTA. To verify that Galardin inhibited caseinolytic activity, the gels were incubated in the MMP substrate buffer with 10 *μ*M Galardin or 10 *μ*M of its structural analogue (negative control). We verified that Galardin does not inhibit caseinolytic activity caused by serine proteinases. Therefore, the serine proteinase plasmin was used and the gels were incubated in the serine proteinase substrate buffer (50 mM Tris-HCl, pH 7.5, 100 mM glycine and 15 mM EDTA) with or without 10 *μ*M Galardin.

### Immunohistochemistry

Deparaffinised sections were incubated for 30 min with 0.3% hydrogen peroxide in methanol and microwave heated in EDTA buffer. Subsequently, an indirect immunoperoxidase technique was applied, using mouse anti-human monoclonal MMP7 antibody (dilution 1/300). Negative controls consisted of omission of the primary antibody.

## RESULTS

### Desmoid CM is able to stimulate invasion

**Figure 1:**
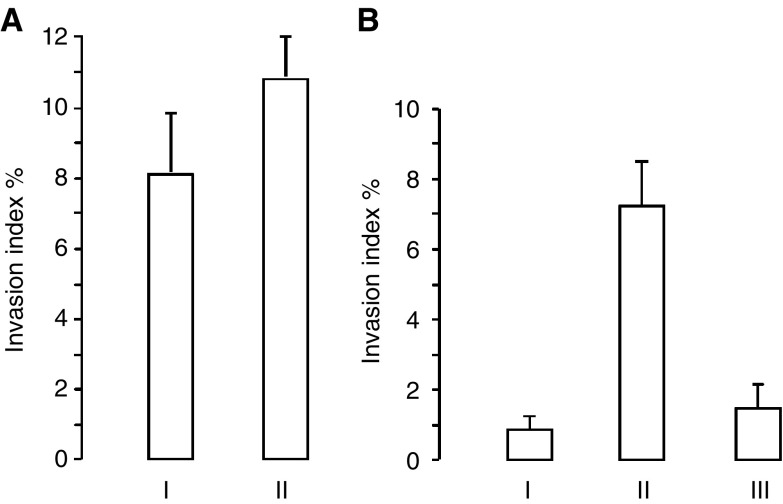
Collagen gel invasion assays with desmoid and HCT-8/E11 cells. (**A**) Desmoid cells were seeded on top of collagen type I gel and incubated with (I) normal medium or (II) CM of desmoid cultures. (**B**) The HCT-8/E11 cells were seeded on top of collagen type I gel and incubated with (I) normal medium; (II) CM of desmoid cultures; (III) CM of fascia cultures. The invasion indexes were calculated as the number of cells inside the gel over the total number of cells. Values are means±s.d. of four different experiments, performed in duplicate.

Desmoids are locally very invasive tumours. To study their invasive character, collagen invasion assays were performed, which showed that desmoid cells were invasive in type I collagen gels (8.0%, s.d. 2.0) and that their invasion capacity could be stimulated by addition of desmoid CM (10.8%, s.d. 1.2) (Figure 1A

). To test the hypothesis that invasion-stimulating soluble factors were present in desmoid CM, collagen invasion assays with HCT-8/E11 colon cancer cells were performed. These colon cancer cells were used as a model because it was previously shown that they were noninvasive in type I collagen, but can become invasive depending on stimulation (Debruyne *et al*, 2002; Regnauld *et al*, 2002; Nguyen *et al*, 2002). In the presence of normal medium (DMEM), no invasion of the cells into the collagen was observed. By replacing DMEM with the 5 × concentrated desmoid CM, an invasion of 6.9% of the HCT-8/E11 cells into the collagen gel (Figure 1B) was observed after 24 h. The experiments were also performed using CM of primary fascia cultures from the same patients. The 5 × concentrated fascia CM had no or minimal invasion stimulatory effects on the HCT-8/E11 cells. Similar results were obtained using the colorectal cancer cell line LoVo. In the presence of normal medium, 2.2% (s.d. 1.1) of the LoVo cells invaded into the collagen. Addition of desmoid CM resulted in an invasion of 9.7% (s.d. 0.8) of the LoVo cells into the collagen gel.

These results suggest the presence of invasion-stimulating soluble factors in the desmoid CM.

### Overexpression of MMPs in desmoid tumours

Affymetrix microarray and Atlas Blots (Clontech) were utilised to screen for genes overexpressed in desmoid tumours compared to normal fascia from the same patients (data not shown). These preliminary data suggested that MMPs were overexpressed and tissue inhibitors of MMPs (TIMPs) underexpressed in desmoid tumour cells.

mRNA expression profiles of MMP1, MMP2, MMP3, MMP11, MMP12, MMP13 and MMP14 in four desmoid *vs* fascia samples were performed by quantitative RT–PCR using specific sRNA as internal standards. The results are expressed as arbitrary units corrected by the internal standard, per unit of 28S. A striking overexpression of MMP1, MMP3, MMP11, MMP12 and MMP13 in the desmoid samples was found, as shown in Table 1

**Table 1:** mRNA levels of MMP1, MMP3, MMP11, MMP12, MMP13, TIMP1 and TIMP3

**Target**	**Tissue**	**Mean value (range)**
MMP1	Desmoid	0.723 (0.187–1.958)
	Fascia	0.131 (0.043–0.237)
		
MMP3	Desmoid	3.980 (1.232–8.301)
	Fascia	0.161 (0.005–0.372)
		
MMP11	Desmoid	0.847 (0.431–1.450)
	Fascia	0.083 (0.024–0.139)
		
MMP12	Desmoid	0.363 (0.029–1.196)
	Fascia	0.003 (0.002–0.003)
		
MMP13	Desmoid	0.285 (0.132–0.633)
	Fascia	0.022 (0.011–0.034)
		
TIMP1	Desmoid	0.600 (0.290–1.038)
	Fascia	1.323 (0.858–2.047)
		
TIMP3	Desmoid	0.642 (0.516–0.897)
	Fascia	1.406 (0.824–1.819)

Values are expressed in arbitrary units per unit of 28S rRNA. Mean value is the average of measurements in four different desmoids and fascia samples.

and Figure 2

**Figure 2:**
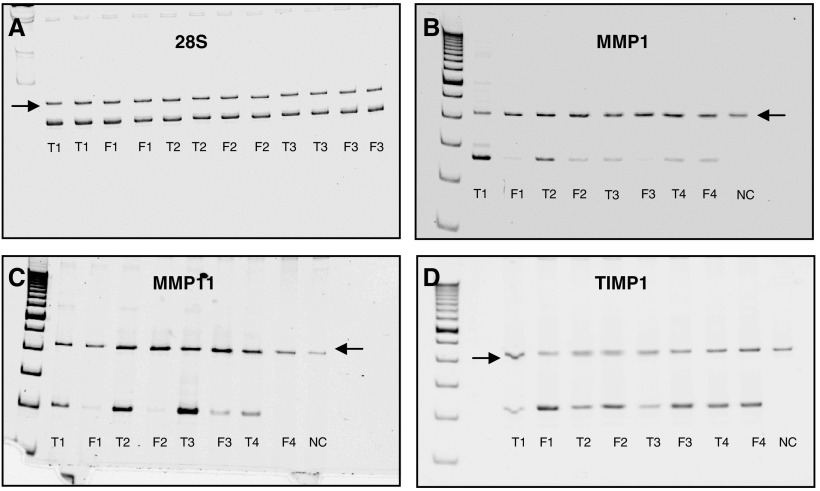
Electrophoretic pattern of RT–PCR amplification products from cellular mRNA and synthetic RNA. Total RNA (10 ng) from four primary desmoid tumour (T) and fascia (F) cultures, or 2 × 10^5^ copies of standard synthetic RNA were submitted to RT–PCR. Arrows indicate the signal obtained for the standard RNA. (**A**) Expression of 28S; (**B**) expression of MMP1; (**C**) expression of MMP11; (**D**) expression of TIMP1. The RT–PCR products had the expected sizes.

. There was no significant difference in the mRNA expression levels of MMP2 and MMP14 between the desmoid and fascia samples in these four cases and according to the microarray results, MMP9 was not expressed above background level in most desmoids (data not shown). For the expression profile of MMP7 in desmoids, TaqMan RT–PCR was used. Results were expressed as the relative expression in desmoids against the corresponding fascia and were normalised for the housekeeping gene PBGD (Table 2

**Table 2:** RT–PCR (Taqman) expression profile of MMP7

	**Patient 1**	**Patient 2**	**Patient 3**	**Patient 4**	**Mean**
MMP7	10.6 : 1	2.6 : 1	62.3 : 1	61.3 : 1	33.9 : 1
					

**Figure 3:**
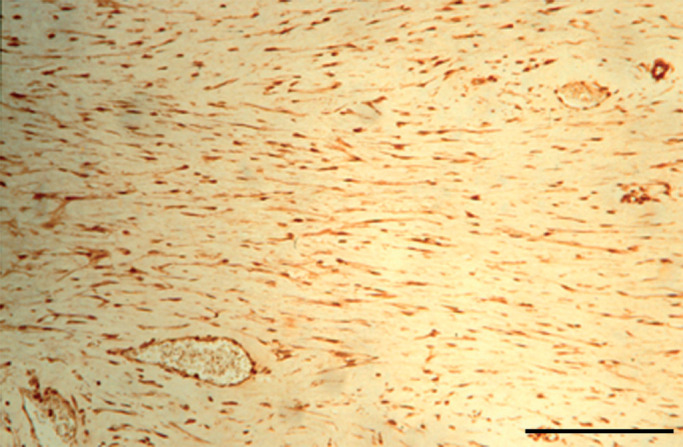
Immunoperoxidase staining of MMP7. The spindle-shaped cells of the desmoid tumour are decorated. The vessel walls are stained as well. Scale bar=100 *μ*m.

Indicative values for the relative expression ratio in desmoids against the corresponding fascia, normalised for the housekeeping gene PBGD. Interindividual variability while high shows the same trend in all four patients.

). Matrix metalloprotein 7 was upregulated (mean average 33.9-fold) in all desmoids tested compared to the corresponding fascia. Immunohistochemistry confirmed MMP7 expression at the protein level in desmoid tumour cells and tumour-associated endothelial cells *in vivo* (Figure 3

). Adipocytes and occasional lymphocytes did not reveal any staining.

**Figure 4:**
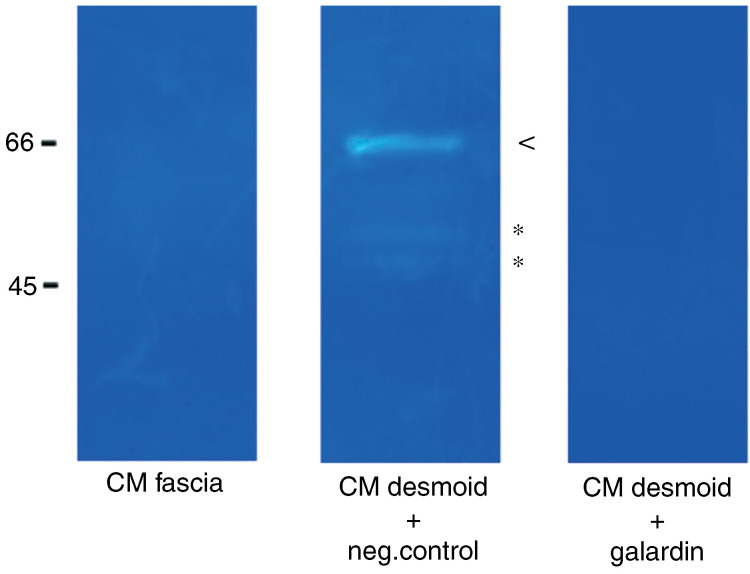
Collected conditioned medium (CM) of desmoids and fascia were analysed by casein zymography without or in the presence of 10 *μ*M Galardin or 10 *μ*M of its negative control. Equal amounts of CM obtained from equal cell numbers were subjected to electrophoresis and the gel was processed as described in Materials and Methods. Molecular size standards are indicated at the left. Stars (^*^) represents active stromelysins, arrowheads (<) represents the prostromelysins. A representative result of three different experiments is shown.

Zymography was used to confirm the overexpression of caseinolytic MMPs or stromelysins at the protein level. The CM of desmoids clearly showed higher caseinolytic activity than the fascia CM (Figure 4

). To verify that the detected caseinolytic activities were specifically derived from MMPs, the gels were incubated with the MMP substrate buffer with 10 mM EDTA. No caseinolytic activity was observed after treatment with EDTA. To verify that Galardin inhibited caseinolytic activity, the gels were incubated in the MMP substrate buffer with 10 *μ*M Galardin. Treatment with Galardin inhibited the caseinolytic activity (Figure 4). Same concentrations of Galardin did not inhibit caseinolytic activity by the serine proteinase plasmin, indicating the specificity of Galardin as an MMP inhibitor (data not shown). These results suggested that the caseinolytic bands in the desmoid CM are due to MMPs that are sensitive to Galardin. In conclusion, the MMP expression profile in desmoid tumours showed an upregulation of especially stromelysins and collagenases, but no significant expression or differential expression of gelatinases.

Since physiologic inhibitors regulate the proteolytic activities of MMPs, the expression levels of TIMPs, TIMP1, TIMP2 and TIMP3 were examined by quantitative RT–PCR using specific sRNA as internal standards. In 75% of the desmoid samples, TIMP1 and TIMP3 levels were significantly downregulated compared to the control samples (Table 1; Figure 2). For TIMP2, the interindividual mRNA expression was variable and no significant difference in expression levels was observed (data not shown).

### Role of MMPs in facilitating invasion

**Figure 5:**
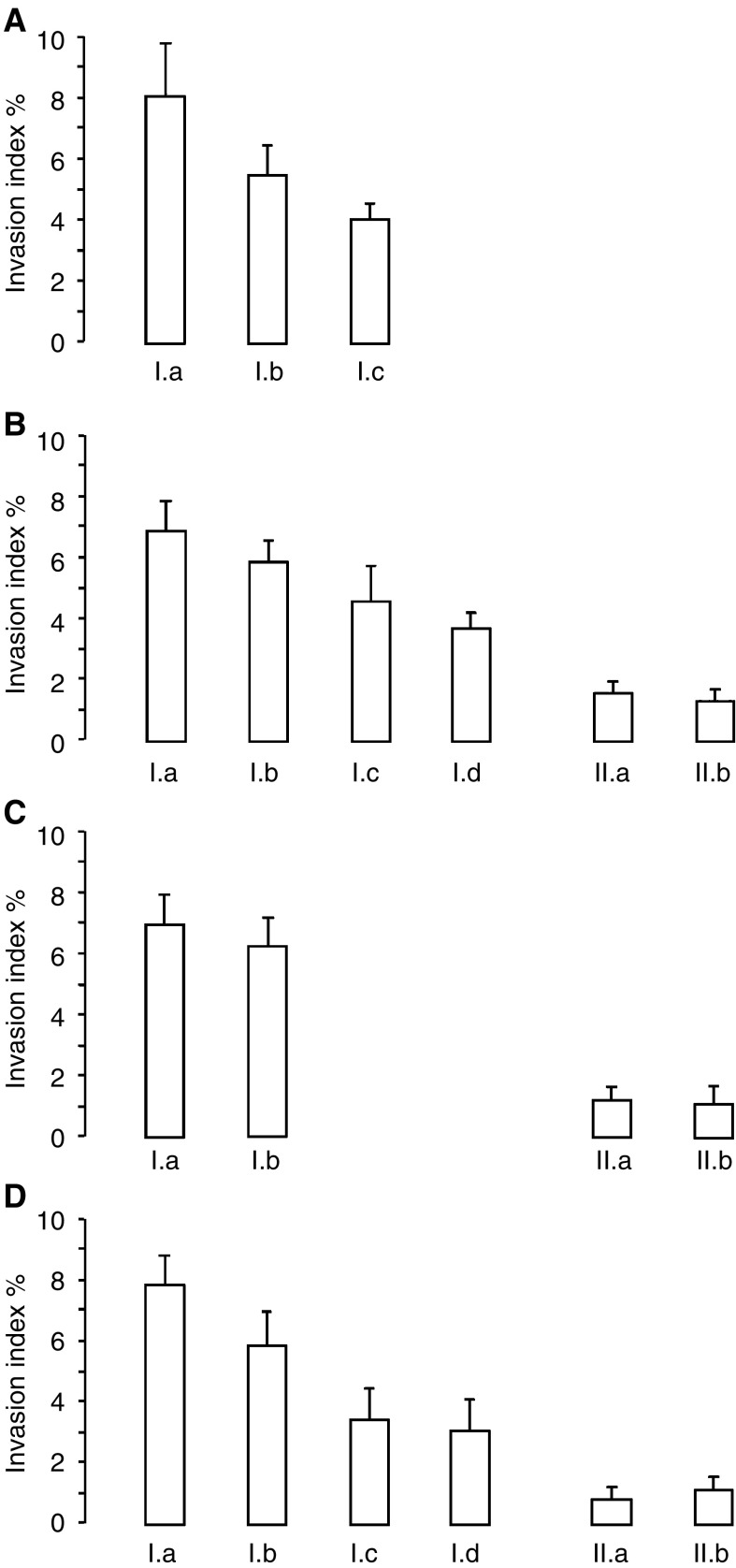
Invasion assays with desmoid and HCT-8/E11 cells, incubated with MMP inhibitors, proteinase inhibitors and/or desmoid or fascia CM. (**A**) Desmoid cells were seeded on top of collagen type I gel and incubated with (I) normal medium, (II) normal medium with addition of Galardin 10 *μ*M, (III) CM of primary fascia cultures. (**B**) HCT-8/E11 cells were seeded on top of collagen type I gel and incubated with: (I) desmoid CM with addition of (Ia) control 10 *μ*M (a structural analogue of Galardin), (Ib) Galardin 0.1 *μ*M, (Ic) Galardin 1 *μ*M and (Id) Galardin 10 *μ*M; (II) CM of fascia cultures with addition of (IIa) control 10 *μ*M (a structural analogue of Galardin and (IIb) Galardin 10 *μ*M. (**C**) HCT-8/E11 cells were seeded on top of collagen type I gel and incubated with: (I) desmoid CM without (Ia) or with addition of aprotinin 10 *μ*g/ml (Ib); (II) fascia CM without (IIa) or with addition of aprotinin 10 *μ*g/ml (IIb). (**D**) Collagen invasion assay with HCT-8/E11 cells incubated with desmoid or fascia CM and addition of different concentrations of a physiologic MMP inhibitor TIMP1. (I) desmoid CM without addition of TIMP1 (Ia), with addition of TIMP1 20 ng/ml (Ib), TIMP1 200 ng/ml (Ic), TIMP1 500 ng/ml (Id). (II) Conditioned medium of fascia cultures without (IIa) and with addition of TIMP1 500 ng/ml (IIb). The invasion indexes were calculated at the number of cells inside the gel over the total number of cells. Values are means±s.d. of four different experiments.

To assess the role of MMPs in facilitating invasion, invasion assays were carried out in the presence of MMP inhibitors. Toxicity of all inhibitors used was tested on proliferation of HCT-8/E11 cells and desmoid tumour cells. None of the inhibitors influenced growth significantly after 24 h of incubation as measured with the sulphorhodamine B assay (data not shown). The invasion capacity of desmoid cells into the collagen gel could be partly inhibited by Galardin (5.7%, s.d. 1.5), a synthetic MMP inhibitor, or by CM obtained from fascia cultures (4.0%, s.d. 0.3) (Figure 5A

), which express higher levels of physiologic MMP inhibitors or TIMPs (see Figure 2). Galardin also dose dependently decreased desmoid CM-stimulated invasion of HCT-8/E11 cells in a type I collagen gel (Figure 5B). At the highest concentration (10 *μ*M), Galardin decreased the invasion of the HCT-8/E11 colon cancer cells by approximately 50%. An inactive structural analogue of Galardin had no significant effect on HCT-8/E11 invasion stimulated by desmoid CM. Addition of Galardin to fascia CM had no marked effect. Aprotinin, a broad-spectrum serine proteinase inhibitor, had only minimal effects on desmoid-stimulated invasion of HCT-8/E11 cells (Figure 5C). There was no additional or synergistic effect of a combination of Galardin and aprotinin on invasion. Similar results were obtained using desmoid cells. To examine the putative inhibitory role of TIMPs on invasion, collagen invasion assays were carried out with the addition of TIMP1. Similarly as Galardin, TIMP1 dose dependently decreased desmoid CM-stimulated invasion of HCT-8/E11 cells in a type I collagen gel (Figure 5D).

**Figure 6:**
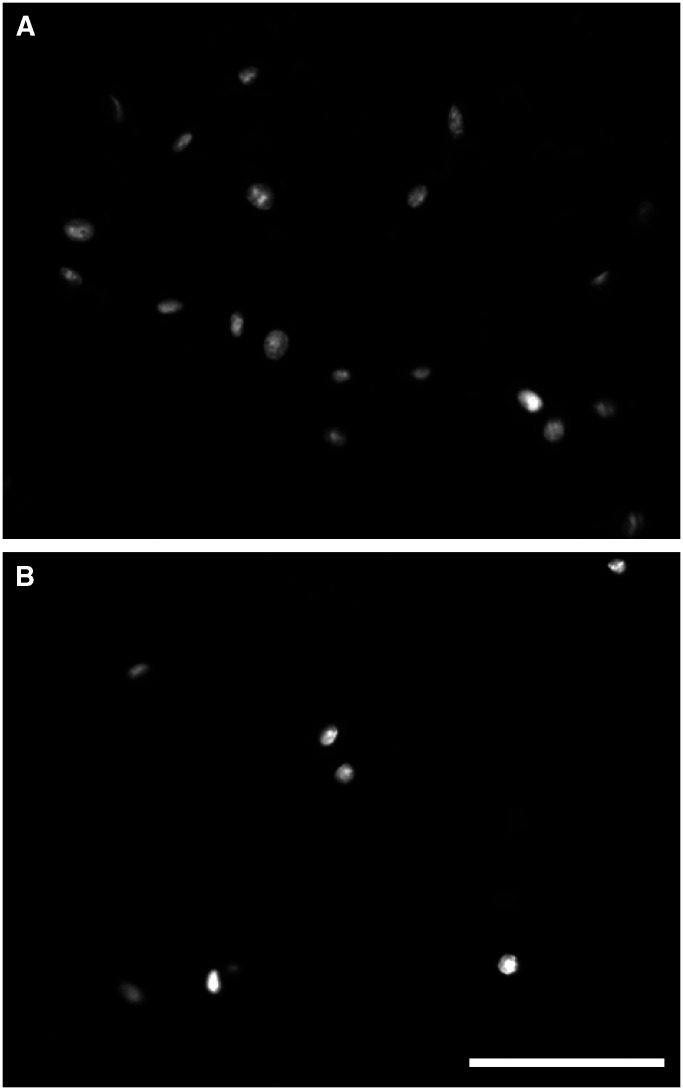
4,6-Diamidino-2-phenylindole-stained cells on the bottom layer of the membrane. There was a significantly higher number of cells that crossed the membrane in cultures treated with a carrier (**A**) than in cells treated with an MMP inhibitor (**B**). There is some background staining around the holes in the membrane. Scale bar=100 *μ*m.

Equal numbers of cells from primary cell cultures derived from eight cases of desmoids and normal fibroblasts (fascia) from the same patients were grown on Matrigel in a modified Boyden chamber. The number of cells invading through the Matrigel was determined by counting the number of cells reaching the lower portion of the permeable membrane. Twice the number of desmoid cells passed through the membrane compared to the number of normal fibroblasts. For the desmoid cases, a mean of 19.5 (s.d. 3.4) cells per hpf passed the membrane. When treated with an MMP inhibitor Ilomastat (=Galardin) the number of desmoid cells passing through the Matrigel decreased to 8.4 (s.d. 2.9) cells per hpf (Figure 6A and B

), which was a significant difference (*P*<0.05).

## DISCUSSION

We found that cells from desmoid tumours express multiple MMPs at higher levels than normal fibroblasts, and that cells from the tumours express TIMPs at lower levels than do cells from normal fibroblasts. Furthermore, desmoid cells produce soluble factors that can stimulate cell invasion, which can be blocked partially by inhibiting MMPs. Together, these data suggest a critical role for MMPs in this locally invasive tumour.

Using quantitative RT–PCR, overexpression of MMP1, MMP3, MMP7, MMP11, MMP12 and MMP13 in desmoid tumours was demonstrated. The overexpression of stromelysins in desmoid tumours at the mRNA level was confirmed at the protein level with zymography. Although zymography results may not always accurately reflect the proteolytic activity of the enzymes *in vivo*, clearly higher caseinolytic activity in desmoid CM was observed. Matrix metalloprotein 7 is commonly expressed by epithelial-derived tumour cells, but immunohistochemical staining confirmed the RT–PCR data and showed that mesenchymal desmoid cells can also express this MMP. Also, vascular endothelial cells in desmoids expressed MMP7, which is similar as in MMP7-positive epithelial tumours (Nagashima *et al*, 1997).

TIMPs can form 1 : 1 stoichiometric complexes with active MMPs, resulting in the inhibition of the enzymatic activity of almost all members of the MMP family (Gomez *et al*, 1997). Therefore, the expression levels of TIMP1, TIMP2 and TIMP3 were examined. In all the tumours analysed, there was a clear underexpression of at least one of the TIMPs; in some tumours there was even a downregulation of all TIMPs examined. An imbalance between activators and inhibitors of the MMPs, resulting in excessive degradation of the ECM has been implicated in tumour invasion (Tanaka *et al*, 1995). The demonstrated lower expression levels of TIMPs in desmoids probably enhance the proteolytic activities of the MMPs. However, the relationship between MMPs and TIMPs in tumour development may be more complex than just an imbalance between MMP and TIMP expression, as TIMPs exhibit additional functions, some of which may directly enhance tumour cell growth.

The increased expression of MMPs in the invasive desmoid tumours and the ability of MMPs to degrade ECM barriers suggested a role for these enzymes in desmoid invasion. In two different experiments, the involvement of MMPs in desmoid invasion could be shown. In the collagen invasion assays with HCT-8/E11 colon cancer cells, desmoid CM treated with a broad-spectrum MMP inhibitor resulted in a 50% decrease of the invasion-stimulating effects of desmoid CM. Similar results were obtained in collagen invasion assays with desmoid cells. The fact that fascia CM also had an inhibitory effect on desmoid invasion is probably due to the higher expression of TIMPs by the fascia cells, as was shown by the invasion experiment with addition of TIMP1.

The Matrigel experiments also showed that the number of desmoid cells that invaded the membrane was decreased significantly when treated with an MMP blocking agent.

Together, these findings suggest that the invasion-stimulating effect of desmoids are partly related to the overexpression of MMPs.

What is activating MMP expression in desmoid tumours? Matrix metalloprotein regulation is complex and occurs at the levels of both gene transcription and protein activation. The MMP genes are transcriptionally responsive to a wide variety of external stimuli, including cytokines, growth factors and changes in cell–cell and cell–matrix interactions. Tumour associated expression of many MMP family members requires the activity of a variety of oncogenic transcription factors, such as AP-1 and Ets (Crawford and Matrisian, 1996). Matrix metalloprotein 7 or matrilysin has been identified as a target gene of *β*-catenin/TCF in colon cancer (Crawford *et al*, 1999). They showed that expression of the PEA3 Ets family, in conjunction with accumulation of *β*-catenin, leads to upregulation of MMP7 gene transcription in colon cancer. In our previous work, we demonstrated *β*-catenin/TCF3-mediated transcriptional activation in desmoid tumours. Therefore, the fact that an upregulation of MMP7 was found in desmoids suggests that it is not only a target of *β*-catenin in epithelial tumours but probably also in mesenchymal tumours. Most of the promoters of the other overexpressed MMPs contain potential TCF binding sites, aside from the AP-1 and ETS binding sites. So it is possible that they may be regulated by *β*-catenin-mediated TCF-dependent transcription, perhaps in combination with other oncogenic transcription factors. During the preparation of this manuscript, two other MMPs, MMP26 and MT1-MMP, were identified in colon cancer as *β*-catenin targets (Takahashi *et al*, 2002; Marchenko *et al*, 2002). The results of MMP overexpression in desmoids, together with the identification of several MMPs as *β*-catenin targets in colon cancer, strongly suggest an important interplay between *β*-catenin signalling, matrix production and matrix remodelling; it also suggests that one of the ways accumulated *β*-catenin contributes to carcinogenesis is by activating transcription of MMP genes.

In the entire process of cancer invasion, the local host tissue microenvironment can be an active participant. Besides the vascular, immune and inflammatory cells, the microenvironment of a tumour consists mainly of myofibroblasts. An important role for myofibroblasts was suggested by the fact that large numbers of myofibroblasts were found near the invasive front of colon cancer or other invasive carcinomas (Nakayama *et al*, 1998). Recently, it became evident that MMPs are mostly produced by stromal cells and not by the cancer cells (Noël *et al*, 2001). The cancer cells can recruit host cells, such as myofibroblasts, to become proteinase factories. Indeed, except for MMP7, the overexpressed MMPs found in the myofibroblastic desmoids, have previously been described as MMPs mainly expressed by the stromal cells. Increased expression of MMP1 has been observed in the stroma around lung carcinomas (Curran and Murray, 1999), squamous cell carcinomas of the head and neck and colorectal carcinomas. Matrix metalloprotein 11 mRNA and protein were found specifically in fibroblastic cells, immediately surrounding carcinoma cells (De Wever and Mareel, 2002). Here, we show that the desmoid tumours themselves can induce the overexpression of their MMPs, without being stimulated or influenced by epithelial cancer cells. The data suggest that the desmoid cells synthesize MMPs via an autocrine mechanism. This is in contrast to epithelial cancers, where myofibroblasts are stimulated in a paracrine manner by the tumour cells to produce MMPs. The fact that myofibroblasts promote invasion of epithelial cancers and can facilitate their own invasion through production of MMPs suggests an important role for these cells in tumour invasion of a variety of tumour types.

Our study shows a role for MMPs, facilitating the ability of cells from desmoids to invade through the extracellular matrix. In combination with data from other tumour types, it suggests a common role for mesenchymal, myofibroblastic cells in regulating cell invasion. Since desmoid tumours are locally invasive, but not metastatic, strategies to slow their invasive behaviour could lead to an effective therapy. Matrix metalloproteinase inhibitors have the potential to act as such therapeutic agents, although further *in vivo* studies would be required before adopting the use of such agents in clinical practice.
